# Supplementary material for: Resolving the full spectrum of human genome variation using Linked-Reads
Source: Genome Res. 2019 Apr;29(4):635–45. doi: 10.1101/gr.234443.118 (PMC6442396; doi:10.1101/gr.234443.118)
Supplement: Supplemental Material [file supp_gr.234443.118_Supplemental_longranger-2.2.zip › longranger-2.2.tar.bz2!/lr2.2-public/lib/stan/cmdstan/stan/doc/api/html/check__lower__triangular_8hpp_source.html]

Stan: src/stan/math/error\_handling/matrix/check\_lower\_triangular.hpp Source File


|  |  |
| --- | --- |
|  | Stan  2.5.0  probability, sampling & optimization |


- Main Page
- Related Pages
- Namespaces
- Classes
- Files

- File List
- File Members

All Classes Namespaces Files Functions Variables Typedefs Enumerations Enumerator Friends Macros Pages

- src
- stan
- math
- error\_handling
- matrix

check\_lower\_triangular.hpp

Go to the documentation of this file.

1 #ifndef STAN\_\_MATH\_\_ERROR\_HANDLING\_\_MATRIX\_\_CHECK\_LOWER\_TRIANGULAR\_HPP

2 #define STAN\_\_MATH\_\_ERROR\_HANDLING\_\_MATRIX\_\_CHECK\_LOWER\_TRIANGULAR\_HPP

3

4 #include <sstream>

5 #include <stan/math/matrix/Eigen.hpp>

6 #include <stan/meta/traits.hpp>

7 #include <stan/math/error\_handling/dom\_err.hpp>

8

9 namespace stan {

10

11  namespace math {

12

26  template <typename T\_y, typename T\_result>

27  inline bool check\_lower\_triangular(const char\* function,

28  const Eigen::Matrix<T\_y,Eigen::Dynamic,Eigen::Dynamic>& y,

29  const char\* name,

30  T\_result\* result) {

31  for (int n = 1; n < y.cols(); ++n) {

32  for (int m = 0; m < n && m < y.rows(); ++m) {

33  if (y(m,n) != 0) {

34  std::stringstream msg;

35  msg << name << " is not lower triangular;"

36  << " " << name << "[" << stan::error\_index::value + m << ","

37  << stan::error\_index::value + n << "]="

38  << "%1%";

39  std::string msg\_string(msg.str());

40  return dom\_err(function,y(m,n),"",msg\_string.c\_str(),"",result);

41  }

42  }

43  }

44  return true;

45  }

46

47  }

48 }

49 #endif

dom\_err.hpp

stan::math::dom\_err

bool dom\_err(const char \*function, const T &y, const char \*name, const char \*error\_msg, const T\_msg error\_msg2, T\_result \*result)

**Definition:** dom\_err.hpp:36

traits.hpp

stan::error\_index::value

**Definition:** traits.hpp:20

Eigen.hpp

stan::math::check\_lower\_triangular

bool check\_lower\_triangular(const char \*function, const Eigen::Matrix< T\_y, Eigen::Dynamic, Eigen::Dynamic > &y, const char \*name, T\_result \*result)

Return true if the specified matrix is lower triangular.

**Definition:** check\_lower\_triangular.hpp:27


---

[ Stan Home Page ]

*© 2011–2014,
Stan Development Team.*
